# Factors associated with anesthetic satisfaction after cesarean delivery under neuraxial anesthesia

**DOI:** 10.1186/s40981-018-0206-x

**Published:** 2018-09-10

**Authors:** Mitsuru Ida, Junko Enomoto, Yumiko Yamamoto, Hiroki Onodera, Masahiko Kawaguchi

**Affiliations:** Department of Anesthesiology, Nara Medical University, 840 Shijo-cho, Kashihara, Nara 634-8522 Japan

**Keywords:** Cesarean delivery, Spinal anesthesia, Patient satisfaction

## Abstract

**Introduction:**

Evaluating patient satisfaction with anesthesia is critical for improving their experiences. We investigated perioperative anesthetic satisfaction and associated predictive factors in patients receiving cesarean delivery under neuraxial anesthesia (spinal anesthesia only or combined spinal-epidural anesthesia).

**Methods:**

This was an institutionally approved retrospective chart review of patients who received cesarean delivery under spinal anesthesia and postoperative evaluation administered by anesthesiologists from January 2009 to December 2013. Multiple pregnancies and patients reporting headache prior to cesarean delivery were excluded. Patients were divided into satisfied and not satisfied groups according to their scores from the 4-point Likert scale. Multivariate analysis was used to identify explanatory factors associated with satisfaction.

**Results:**

Of 813 patients enrolled, 425 (52.2%) were classified as satisfied. Combined spinal–epidural anesthesia (CSEA) (odds ratio, 3.3; 95% confidence interval, 1.08–10.1) was positively associated with satisfaction. Paresthesia during needle insertion (odds ratio, 0.56; 95% confidence interval, 0.42–0.76), lightning pain during neuraxial anesthesia (odds ratio 0.62; 95% confidence interval, 0.39–0.98), failed block (odds ratio 0.28; 95% confidence interval, 0.09–0.87), and intraoperative use of antiemetic (odds ratio 0.71; 95% confidence interval, 0.53–0.94) were negatively associated with satisfaction. In the 792 patients receiving spinal anesthesia only, the same factors except for CSEA were associated with satisfaction.

**Conclusions:**

The addition of epidural to spinal anesthesia for cesarean delivery can increase patient satisfaction, whereas paresthesia during needle insertion, lightning pain, failed block, and the use of intraoperative antiemetic were major obstacles to patient satisfaction.

## Background

Patient-reported outcomes have become a core indicator of medical quality in addition to objective measures of procedural safety and efficacy [1]. There have been many studies on procedural safety and efficacy for cesarean delivery, such as investigations on the benefits of ultrasonography for obese pregnancy [2] and prevention of hypotension after spinal anesthesia [3]. In contrast, there is still much room for improvement of patient-centered anesthesia outcomes. A recent observational study reported that excess body weight, induced labor, and need for anesthesia top-up were associated with dissatisfaction during epidural labor [4]. There have been few studies, however, on factors related to anesthesia satisfaction among patients undergoing cesarean delivery [5, 6].

It is widely accepted that avoidance of general anesthesia improves the safety of cesarean delivery, so further advances in medical quality and patient experience require more effective spinal anesthesia. Therefore, we investigated postoperative anesthesia satisfaction and associated factors, particularly factors associated with anesthesia management.

## Methods

This was a retrospective observational study approved by the Nara Medical University Institutional Review Board, Kashihara, Nara, Japan (Chairperson Prof. M Yoshizumi, Approval No. 1480 on February 20, 2017). Patients who underwent cesarean delivery under spinal anesthesia and a postoperative evaluation by anesthesiologists at Nara Medical University between January 2009 and December 2013 were considered candidates. Patients were excluded because of missing data, multiple pregnancies, headache prior to cesarean delivery, or conversion from spinal to general anesthesia during surgery.

In our hospital, extreme emergency cesarean delivery (stat cesarean delivery) is performed on patients under general anesthesia. Therefore, anesthesiologists are usually able to explain the single-shot spinal techniques as well as the side effects and complications associated with spinal anesthesia to patients. In case a patient requires epidural catheter placement, anesthesiologists explain the advantages and disadvantages of epidural catheter placement.

An intravenous catheter was inserted before arrival at the operating theater. Patients were then placed in the supine position for attachment of standard anesthesia monitors. Spinal anesthesia was performed by using a 25-G Quincke needle via the L3-4 or L4-5 interspace following lidocaine infiltration in the lateral position. Anesthesia was provided with 10–15 mg hyperbaric bupivacaine 0.5%, 100 μg morphine, and 10 μg fentanyl. Anesthesiologists usually explain to manage postoperative pain with intrathecal morphine. However, some pregnant women searched the way of postoperative pain relief before visiting anesthetic clinic. In case that patients requested epidural anesthesia, we made it consistent with patient’s intentions. When patients requested epidural anesthesia, an epidural catheter was inserted via the L1-2 or L2-3 interspace before spinal anesthesia. Intraoperative management, such as management of fluid status and blood pressure, was at the discretion of each anesthesiologist. After the operation, patients with an epidural catheter began epidural analgesia (0.2% ropivacaine) under the following conditions: bolus 5 mL, continuous infusion 4 mL/h, and lockout 60 min.

From 2 to 7 days after delivery and before discharge, anesthesiologists queried patients about paresthesia during needle insertion and lightning pain during spinal and/or epidural anesthesia, postpartum headache, and postoperative nausea, vomiting, pain, numbness of lower limbs, and itching with two choices “yes” or “none.” “Lightning pain” was defined as sudden and sharp pain accompanying needle puncture, and “paresthesia” was defined as an uncomfortable pain accompanying needle. Patients were then asked to evaluate overall anesthetic satisfaction by using a 4-point Likert scale (satisfaction, neutral, mild dissatisfaction, and dissatisfaction).

Additional information regarding maternal demographics (age, height, weight, parity, gestational weeks, and comorbidity), intraoperative data (surgical duration, time of day, block height, failed block, intraoperative use of sedatives or analgesics, and intraoperative use of antiemetic), neonatal Apgar score at 5 min postdelivery, and neonatal weight were collected from electronic medical records. The operative time of day was divided into four categories: daytime elective surgery, daytime emergency surgery, nighttime emergency surgery, and surgery on holidays. A failed block was defined as the need to repeat the spinal technique. The block height was divided into more than or equal to the level of Th4 or not.

### Statistical analysis

Data are presented as the mean (standard deviation) or number. Univariate and multivariate logistic regression analyses were used to identify factors associated with perioperative anesthetic satisfaction. We defined the satisfied group as those patients who answered “satisfaction” to the question on the 4-point Likert scale. Univariate analysis was performed by using the chi-square test, Fisher’s exact test, Mann–Whitney *U* test, or unpaired *t* test, as appropriate. All explanatory factors were included in multivariate logistic regression analysis. Discrimination of the final models for the satisfied and dissatisfied groups was assessed by using the likelihood ratio test. Calibration of the models was tested by using the Hosmer–Lemeshow test. The area under the receiver operating characteristic curve was computed as a descriptive tool for measuring model bias. In the first step, we analyzed all patients (receiving spinal anesthesia only and combined spinal–epidural anesthesia (CSEA)). In the second step, patients undergoing spinal anesthesia only were analyzed in the same way to eliminate the effects of epidural anesthesia. All data were analyzed by using SPSS version 22.0 (IBM Inc., Armonk, NY, USA), and *p* values of &lt; 0.05 (two-tailed) were considered as indicative of statistical significance.

## Results

**Fig. 1:**
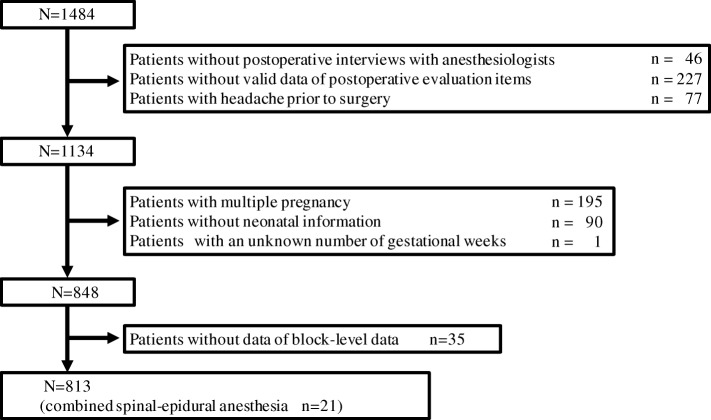
Patient flowchart

A total of 813 pregnant patients meeting the inclusion criteria were enrolled (Fig. 1). Patient demographics are shown in Table 1. Twenty-one patients underwent CSEA without incidence of accidental dural puncture. Eighteen patients underwent repeated spinal anesthesia, and 451 patients experienced paresthesia during needle insertion. Intraoperative use of an antiemetic was used in 348 patients. Postoperative interviews were conducted an average of 4.3 days postdelivery before discharge. Postpartum headache was reported by 109 patients, but no patients needed an epidural blood patch. Lightning pain during neuraxial anesthesia was reported by 96 patients, but none had difficulty walking or required long-term follow-up. In the satisfaction evaluation, 425 (52.2%) patients answered “satisfaction,” 356 (43.7%) “neutral,” 25 (3.0%) “mild dissatisfaction,” and 7 (0.8%) “dissatisfaction.”

**Table 1:** Univariate analysis between the satisfied and not satisfied groups

	All patients (*n* = 813)		*p* value	Spinal anesthesia patients (*n* = 792)		*p* value
	Satisfied group	Not satisfied group		Satisfied group	Not satisfied group	
	*n* = 425	*n* = 388		*n* = 408	*n* = 384	
Maternal demographics						
Age (year)	32.8 (5.1)	32.2 (5.0)	0.13	32.7 (5.2)	32.2 (5.0)	0.17
Body mass index (kg/m^2^)	24.7 (4.4)	24.8 (4.8)	0.75	24.8 (4.4)	24.8 (4.8)	0.92
Number of nulliparous	245	222	0.9	234	219	0.92
Gestational week (weeks)	37.3 (2.6)	37.2 (2.9)	0.87	37.2 (2.7)	37.3 (2.9)	0.75
Comorbidity	106	109	0.3	104	109	0.35
Factors associated with surgery						
Surgical duration (min)	56.3 (16.8)	56.5 (17.0)	0.87	56.3 (16.9)	56.4 (16.7)	0.88
Time zone of surgery			0.86			0.74
Daytime elective surgery	233	216		221	214	
Surgery on holiday	41	38		41	38	
Nighttime emergency surgery	54	44		53	43	
Daytime emergency surgery	97	90		93	89	
Transfusion	20	14	0.43	20	13	0.28
Factors associated with neuroxial anesthesia						
Intraoperative factors						
Epidural anesthesia	17	4	0.002	0	0	NA
Paresthesia during needle insertion	203	248	&lt; 0.001	195	247	&lt; 0.001
Lightning pain	37	59	0.0041	36	59	0.0046
Failed block	4	14	0.0098	4	14	0.011
Block height (Th ≤ 4)	362	314	0.1	346	311	0.15
Sedatives or analgesics	101	114	0.06	98	113	0.08
Anti-emetic	164	184	0.011	162	183	0.019
Postoperative factors						
Nausea and vomiting	98	112	0.05	95	110	0.81
Postoperative pain	352	333	0.24	341	330	0.35
Numbness	5	10	0.13	4	10	0.083
Postpartum headache	45	64	0.013	44	64	0.015
Itching	160	147	0.94	149	146	0.47
Neonatal information						
Apgar score at 5 min	9.6 (0.8)	9.6 (1.0)	0.89	9.6 (0.8)	9.6 (1.0)	0.78
Baby’s weight (g)	2669 (640)	2692 (679)	0.62	2656 (640)	2687 (680)	0.49

Data are presented as the mean and standard deviation in parentheses or as a number; *NA* not available

### Factors associated with satisfaction

Univariate analysis revealed significant associations of satisfaction with CSEA, paresthesia during needle insertion, lightning pain during neuraxial anesthesia, failed block, intraoperative use of antiemetic, and postpartum headache. In the 792 patients who received spinal anesthesia only, the same factors except for CSEA were associated with satisfaction (Table 1). Multivariate analysis revealed that CSEA [odds ratio (OR), 3.3], paresthesia during needle insertion (OR, 0.56), lightning pain during neuraxial anesthesia (OR, 0.62), failed block (OR, 0.28), and intraoperative antiemetic (OR, 0.71) were independently associated with decreased satisfaction. In the patients receiving only spinal anesthesia, the same factors except for CSEA were associated with satisfaction (Table 2).

**Table 2:** Multivariate logistic regression model for the satisfied group

	Odds ratio	95% CI	*p* value
All patients (*n* = 813)			
CSEA	3.3	1.08–10.1	0.035
Paresthesia during needle insertion	0.56	0.42–0.76	&lt; 0.001
Lightning pain	0.62	0.39–0.98	0.043
Failed block	0.28	0.09–0.87	0.028
Antiemetic	0.71	0.53–0.94	0.019
Spinal anesthesia patients (*n* = 792)			
Paresthesia during needle insertion	0.55	0.41–0.74	&lt; 0.001
Lightning pain	0.62	0.39–0.97	0.04
Failed block	0.28	0.09–0.87	0.028
Antiemetic	0.71	0.53–0.95	0.021

In all patients, discrimination of the final model assessed by the likelihood ratio test was significant (*p* &lt; 0.001). The Hosmer–Lemeshow test did not reject a logistic regression model fit (*p* = 0.96). The explanatory model based on these variables had an area under the receiver operating characteristic curve of 0.62 (95% CI, 0.58–0.66). No value exceeded the expected value by 3 ± standard deviation

In the spinal anesthesia patients, discrimination of the final model assessed by the likelihood ratio test was significant (*p* &lt; 0.001). The Hosmer–Lemeshow test did not reject a logistic regression model fit (*p* = 0.96). The explanatory model based on these variables had an area under the receiver operating characteristic curve of 0.62 (95% CI, 0.58–0.66). No such value exceeded the expected value by 3 ± standard deviation

*CSEA* combined spinal–epidural anesthesia, *CI* confidence interval

Post hoc power calculations were performed for multivariate logistic regression models in both groups. For logistic regression analysis, it is recommended that the number of events per explanatory variable be ≥ 10 [7]. The minimum number required to determine associated factors was calculated to be 460 for the 24-variable model that included 425 satisfied patients (of 813) who received either spinal anesthesia alone or CSEA and to be 466 patients for the 24-variable model that included 408 satisfied patients (of 792) who received spinal anesthesia only. Thus, our sample size was sufficient to build the models.

## Discussion

In our study cohort of 813 cesarean delivery patients, 52% were satisfied with anesthesia. Further, epidural catheter placement was positively associated with patient satisfaction. On the other hand, paresthesia during needle insertion, lightning pain during neuraxial anesthesia, failed block, and intraoperative use of antiemetic were negatively associated with patient satisfaction.

Previous studies that evaluated maternal satisfaction regarding spinal anesthesia by using a 2-point scale (satisfaction or dissatisfaction) had high satisfaction rates of 97% [5]. This finding might be explained by the different evaluation methods and the fact that we used a summed satisfaction rate of the patients who answered “satisfaction” plus those who answered “neutral,” which was as high as 95.4%. Other study comparing the satisfaction of pregnant women undergoing cesarean delivery with spinal anesthesia or epidural anesthesia showed that patients underwent cesarean delivery with epidural anesthesia had high score in detailed questionnaire due to low occurrences of pruritus [8]. In our study, the number of patients undergoing cesarean delivery with CSEA was limited and detailed examination was difficult. In previous studies, Likert scale or questionnaire has been used to evaluate maternal satisfaction [5, 8, 9], although there are few valid questionnaires to measure maternal satisfaction in Japan and further study should be needed to make a Japanese version of such questionnaires.

In our study, CSEA was an independent factor associated with increased patient satisfaction, but postoperative pain was not despite occurring frequently in both groups. Enhanced satisfaction may arise from perceived control over treatment decisions because, in our institution, epidural anesthesia is performed only if requested.

Our results showing that paresthesia during needle insertion, lightning pain, and failed block were significantly associated with patient satisfaction were consistent with the results of a prospective study, including both obstetric patients and non-obstetric patients, that evaluated factors associated with patient satisfaction regarding spinal anesthesia [9]. Although the full scope of reasons for lightning pain during neuraxial anesthesia has not been systematically studied, decreased satisfaction may be associated with pain from pressure or stretch receptor activation in the lumbar meninges during dural puncture [10]. It was not surprising that the patients with failed block and who received an intraoperative antiemetic were not satisfied. In patients who experience difficulty during spinal anesthesia, treatment by experienced anesthetists or the use of ultrasonography to reduce the number of punctures may be a better strategy [11]. Intraoperative nausea and vomiting can be caused by cerebral and gut hypoperfusion related to maternal hypotension that stimulate the vomiting center in the brainstem and cause serotonin release, respectively [12, 13]. The prophylactic metoclopramide might provide enhanced patient management [14].

Postpartum headache was associated with reduced satisfaction in the univariate analysis, but this association was not observed in the multivariate analysis. A prospective cohort study found that 39% of women experienced postpartum headache, but 96% of these patients were still able to take care of themselves and their babies [15]. Therefore, we suggest that postpartum headache was not associated with anesthetic satisfaction in the multivariate analysis because it is not a hindrance to maternal care.

Our study had several limitations. As with other retrospective studies, it is possible that important factors associated with patient satisfaction were not included in the patient records. Second, the opioids used for perioperative pain management were not analyzed in detail. However, opioid-induced complications, such as itching and postoperative nausea and vomiting, are more likely to affect patient satisfaction.

## Conclusions

In conclusion, we performed a retrospective study to assess postoperative anesthesia satisfaction and identify factors, particularly anesthesia management factors, associated with pregnant women who underwent cesarean delivery under spinal anesthesia. It is implied that the addition of epidural to spinal anesthesia for cesarean delivery may increase patient satisfaction. However further study is needed to see if this is true or not.

## Abbreviations

CSEA: Combined spinal–epidural anesthesia
OR: Odds ratio
